# Evaluation of methods and marker Systems in Genomic Selection of oil palm (*Elaeis guineensis* Jacq.)

**DOI:** 10.1186/s12863-017-0576-5

**Published:** 2017-12-11

**Authors:** Qi Bin Kwong, Chee Keng Teh, Ai Ling Ong, Fook Tim Chew, Sean Mayes, Harikrishna Kulaveerasingam, Martti Tammi, Suat Hui Yeoh, David Ross Appleton, Jennifer Ann Harikrishna

**Affiliations:** 1 Biotechnology & Breeding Department, Sime Darby Plantation R&D Centre, 43400 Serdang, Selangor Malaysia; 2 Institute of Biological Sciences, University Malaya, 50603 Kuala Lumpur, Malaysia; 3 Department of Biological Sciences, National University of Singapore, Singapore, 117543 Singapore; 4 School of Biosciences, University of Nottingham, Sutton Bonington Campus, Nr, Loughborough, LE12 5RD UK; 5 Centre of Research in Biotechnology for Agriculture (CEBAR), University of Malaya, 50603 Kuala Lumpur, Malaysia

**Keywords:** Genomic prediction, Complex traits, Machine learning, Predictive modeling, Marker-assisted selection, SSR, SNP, Perennial crop

## Abstract

**Background:**

Genomic selection (GS) uses genome-wide markers as an attempt to accelerate genetic gain in breeding programs of both animals and plants. This approach is particularly useful for perennial crops such as oil palm, which have long breeding cycles, and for which the optimal method for GS is still under debate. In this study, we evaluated the effect of different marker systems and modeling methods for implementing GS in an introgressed *dura* family derived from a Deli *dura* x Nigerian *dura* (Deli x Nigerian) with 112 individuals. This family is an important breeding source for developing new mother palms for superior oil yield and bunch characters. The traits of interest selected for this study were fruit-to-bunch (F/B), shell-to-fruit (S/F), kernel-to-fruit (K/F), mesocarp-to-fruit (M/F), oil per palm (O/P) and oil-to-dry mesocarp (O/DM). The marker systems evaluated were simple sequence repeats (SSRs) and single nucleotide polymorphisms (SNPs). RR-BLUP, Bayesian A, B, Cπ, LASSO, Ridge Regression and two machine learning methods (SVM and Random Forest) were used to evaluate GS accuracy of the traits.

**Results:**

The kinship coefficient between individuals in this family ranged from 0.35 to 0.62. S/F and O/DM had the highest genomic heritability, whereas F/B and O/P had the lowest. The accuracies using 135 SSRs were low, with accuracies of the traits around 0.20. The average accuracy of machine learning methods was 0.24, as compared to 0.20 achieved by other methods. The trait with the highest mean accuracy was F/B (0.28), while the lowest were both M/F and O/P (0.18). By using whole genomic SNPs, the accuracies for all traits, especially for O/DM (0.43), S/F (0.39) and M/F (0.30) were improved. The average accuracy of machine learning methods was 0.32, compared to 0.31 achieved by other methods.

**Conclusion:**

Due to high genomic resolution, the use of whole-genome SNPs improved the efficiency of GS dramatically for oil palm and is recommended for *dura* breeding programs. Machine learning slightly outperformed other methods, but required parameters optimization for GS implementation.

**Electronic supplementary material:**

The online version of this article (10.1186/s12863-017-0576-5) contains supplementary material, which is available to authorized users.

## Background

Genomic selection (GS) is a form of marker assisted selection (MAS) using markers distributed across the entire genome. Unlike conventional MAS, GS does not require *a priori *association between marker and trait generated from linkage mapping and genome-wide association study (GWAS). Genomic selection was first implemented successfully in cattle, doubling the rate of genetic gain in breeding programs [1, 2]. This method has recently been adapted for crops, including maize, wheat and rice [3, 4]. In commercial perennial crops, the application of GS has been proposed for the selection of complex quantitative traits, including oil yield related traits [5].

As the most efficient oilseed crop in the world, oil palm is able to produce up to 10 times more than other leading oil crops, and has surpassed soy oil since 2008 [6] as the world’s most traded oil. Oil produced in the kernel and mesocarp is suitable for both human consumption and for oleo-chemical industries. The commercial oil palm planting material in Southeast Asia is mainly derived from Deli *dura* x AVROS *pisifera* crosses, selected for high oil yield. The potential reduction of genetic variation in the Deli *dura* due to founder effects is a concern for oil palm breeders. In addition, self-pollination (“selfing”) and sib-mating, which are commonly practiced to concentrate the desired agronomical traits in Deli *dura* populations, has further reduced the genetic variation. This has resulted in various symptoms of inbreeding depression, such as abortive bunch formation, poor fruit set and oil yield depression in Deli *dura* trials [7]. To address this problem, Sime Darby Plantation R&D adopted introgression using *dura* populations acquired from Nigeria to widen the genetic base of the commercial Deli *dura* materials. Deli x Nigerian progenies have improved fresh fruit bunch (FFB) and oil-to-bunch ratio (O/B) values, with useful trait variation observed, resulting in a new series of mother palms for future commercial material. Still, the emphasis placed on the introgression program was low because of the slow nature of breeding progress in oil palm, which is typically 10–12 years per selection cycle [8]. Hence, the ultimate goal of GS is to expedite the breeding progress by maximizing the genetic gains per generation.

In oil palm, GS was first evaluated using simulated data and a limited number of QTLs [9]. Another study using SSRs supports the potential of this method in an oil palm population with narrow genetic base [10, 11]. Although SSRs are informative, the emergence of high-throughput sequencing, which can detect genetic variations across the whole genome, has shifted the preference of marker system towards SNPs. This is because SNPs are generally abundant in the genome and require less time and cost to genotype. As an example, the Malaysian Palm Oil Board (MPOB) has reported of several SNP loci capable of distinguishing different fruit forms in oil palm, which is a monogenic trait [12, 13]. For polygenic traits, including O/B and oil content in mesocarp, genome-wide markers are required to locate the multiple genetic components of these quantitative traits. An example is the mesocarp oil content trait, with multiple QTLs, mainly found in Chromosome 5 [14].

A whole-genome OP200K SNP genotyping array [15] capable of representing genomic information of main breeding stock was developed by referring to the published 1.8-Gb diploid genome of oil palm [16]. This, together with the reduction in genotyping cost, has made GS feasible in oil palm. Even though the use of these genome-wide SNPs have also been proven to work in trait prediction of *tenera* progenies [17], the selection of mother *dura* palms is of more importance for oil palm breeding programs. Before implementation of GS in oil palm *dura* breeding programs, it is crucial to evaluate different marker systems and GS modeling methods. For this purpose, a full-sib family, consisting of 112 individuals, derived from Deli x Nigerian origin was selected for development and assessment of GS models. In total, eight methods, including two machine learning approaches, and two marker systems, SSRs and SNPs were evaluated. Prediction models built for the important yield-related traits were assessed. In general, individual populations available for introgression or further crossing are often small in oil palm. The development of accurate models to guide future crossing and introgression is essential for efficiency development of traits into commercially-relevant parental stock.

## Methods

### Plant materials and phenotyping

Unopened spear leaves from 112 twenty-year-old palms derived from a Deli x Nigerian family, planted in a randomized complete block design trial and maintained at Sime Darby Plantation R&D, Malaysia were sampled [18]. The four years (4th to 8th year) yield, bunch analysis and vegetative measurements were recorded based on the standard industry protocols [19] with modification [20]. From these records, six important oil yield-related traits (fruit-to-bunch (F/B), shell-to-fruit weight (S/F), kernel-to-fruit weight (K/F), mesocarp-to-fruit weight (M/F), total oil yield per palm (O/P) and oil weight-to-dry mesocarp weight (O/DM)) were selected for study. All assayed traits for this study were measured in percentage (%) with the exception of O/P, which was in kg/palm/year. Other traits were measured directly besides O/P. The product of FFB and O/B was used to calculate O/P (FFB * O/B). O/B itself was calculated based on O/DM * DM/WM * M/F * F/B, where DM/WM is the dry matter content of the mesocarp [21]. The phenotype data were averaged across 4 years. Phenotypic analysis was done using a modified “chart” function from the library “PerformanceAnalytics” [22] under R version 3.0.0.

### SSR and SNP genotyping

Total genomic DNA was isolated from 100 mg of each leaf sample using the DNAeasy Plant Mini Kit (Qiagen, Germany). For SSR genotyping, 135 markers from our previous study were selected [18]. In the same study, we have shown that these SSRs, from an average of 10 cM mapping interval provided sufficient power for QTL detection. The SSR amplification was carried out using a M13-tailed forward primer with a four-color fluorescent detection technique [23, 24]. The SSRs with more than two alleles were interpreted as two or more markers in order to convert all genotype values to −1, 0 or 1, an approach similar as published [10], without loss of information. Therefore, the informative SSRs used in this study were represented as 221 markers.

SNP genotyping on the same family was carried out using the OP200K array (170,860 SNPs) [15]. The process was done on the Infinium iScan platform (Illumina Inc., San Diego, CA) according to the manufacturer’s recommendations. The raw intensity SNP data was analyzed and auto-clustered using GenomeStudio version 20,011.1 (Illumina Inc., San Diego, CA) with genotyping module version 1.8.4. A total of 46,933 SNPs were identified to be polymorphic. These genotypes were coded into −1 (AA), 0 (AB) and 1 (BB) format. Missing genotype data were imputed using the na.roughfix function of the *randomForest* package [25] in R.

### Genomic heritabilities and kinship coefficient estimates

Genomic heritabilities for the traits were calculated using an in-house R script, based on a linear model using all informative SNP markers [17, 26, 27]. The kinship coefficients among the individuals was estimated with the R package *related* [28] using 5000 SNPs selected randomly from the full dataset, with estimation method based on Li [29].

### GS methods

This study was performed using an IBM System ×3850 X5 HPC, with 40 Intel (R) Xeon (R) CPU E7–8850 @2.00 GHz processors and 1 Tb of RAM. The methods selected to perform GS in oil palm were RR-BLUP [30], Bayes A [31], B [32],Cπ [32], LASSO [33], Ridge Regression [34]) and two machine learning methods (Support Vector Machine (SVM) and random forest (RF)) [25]. All the methods mentioned were carried out with R version 3.0.0.

RR-BLUP assumes that the marker effects are normally distributed and that they explain the same amount of variance [31]. Bayes A and Bayes B allow for different variance across different markers. Bayes A models the variance using a scaled inverted chi-square distribution [31]. The additional feature of Bayes B compared to A is that it allows for the variance of markers to be zero with a probability of π [32]. The later Bayesian methods are Cπ, Lasso and Ridge Regression: Cπ assumes that π follows a uniform distribution [32]; Bayesian Ridge Regression, on the other hand, uses a Gaussian prior and Lasso [34] uses a Double-Exponential prior [33]. For both SVM and Random Forest, no assumptions were made regarding the markers.

For all methods, a 5-fold cross validation was carried out across all traits, where a single subsample was used as the validation set, and the remaining four samples were used as the training set. This step was carried out until all subsamples had been used for both training and validation steps. RR-BLUP was implemented using the R package *rrBLUP* [30], and Bayesian methods using the *BGLR* package [35]. For Bayesian methods, the number of inner iterations was optimized based an increasing iteration approach using Bayes A. For all traits, the optimization graph became plateau before reaching 10000, indicating that this number of inner iterations was more than sufficient for the estimation of marker effects. The optimization graphs of S/F and O/P were selected for illustration purpose (Additional file 1: Figure S1). In addition, the first 2000 iterations were used as “burn-in”. The same conditions were extended for all Bayesian methods. For Bayesian LASSO, the lambda parameter was set to 25, type set as gamma, rate parameter as 1e^−4^ and shape parameter as 0.55.

SVM was carried out using R package *e1071*. Optimization of the parameters was carried out for each iteration during cross validation using the tune.svm function. Within the training set, another 10-fold cross-validation was used to estimate the optimal parameters. The gamma and cost factors for the SSR dataset were optimized using an exponential range of 2^−15^ to 2^15^ [36], epsilon from 0 to 0.2 under the kernels of “linear”, “radial”, “polynomial” and “sigmoid”. For SNP-based prediction, the gamma and cost factors were set from 2^−30^ to 2^15^. Random forest was carried out using the *randomForest* package [25]. Optimization of the parameters used the *mlr* library for each cross validation step. Like SVM, 10-fold cross validation was used on the training set. The range of mtry optimization range was set from 1 to 221, nodesize from 1 to 50 and ntree was from 500 to 1500 for SSRs. For SNPs, the mtry optimization range was set at 1 to 500, ntree was set from 500 to 3000.

For each iteration, accuracy was defined as the predictive ability, which was calculated as the Pearson correlation coefficient of predicted trait value of the validation set, versus its observed trait value. The overall accuracy was calculated as the mean of the predictive abilities from all iterations. Genomic selection was performed using SSRs, followed by SNPs. Representative regression boxplots were generated for each trait using SNPs for the best method. In order to test for linear assumption made under RR-BLUP and Bayesian models, residuals were calculated from the difference between the predicted trait values and the observed trait values. The residuals were then standardized using the “rstandard” function in R. The residual and Quantile-quantile (QQ) plot were generated using the “plot” and the “qqnorm” functions.

## Results

### Phenotypes, genomic heritabilities and kinship coefficient estimates between individuals

**Fig. 1:**
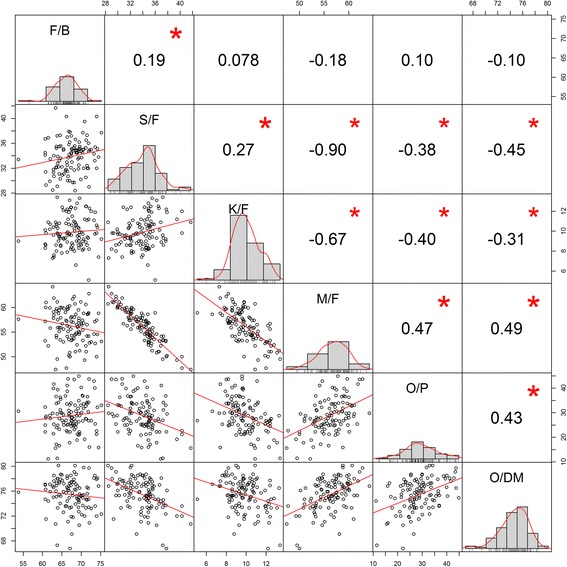
Plot representing phenotypic distribution and correlation for the traits of F/B (%), S/F (%), K/F (%), M/F (%), O/P (kg/palm/year) and O/DM (%) in the Deli x Nigerian family. Diagonal of the plot shows the histograms and the distribution of the observed phenotypes values. The lower off-diagonal is the scatterplot between traits, whereas the upper off-diagonal represents the correlation value between traits. Significant correlations are tagged with the asterisk (*) symbol

**Fig. 2:**
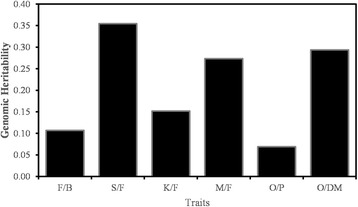
Bar plot showing the genomic heritabilities for the traits of F/B, S/F, K/F, M/F, O/P and O/DM in the Deli x Nigerian family. S/F and O/DM had the highest heritability, followed by M/F, K/F, F/B and O/P

The 4-year recordings for each trait were averaged, with the mean for F/B being 67.37 (±3.74), S/F 33.89 (±2.61), K/F 9.89 (±1.55), M/F 56.23 (±3.37), O/P 28.84 (±7.00) and O/DM 75.44 (±2.54). Since these traits were all yield related, many of them correlated with each other (Fig. 1). In particular, M/F was negatively correlated with S/F and K/F. O/P and O/DM were positively correlated with M/F. The genomic heritabilities of all these traits are shown in Fig. 2. S/F (0.35) and O/DM (0.29) have the highest heritability, followed by M/F (0.27), K/F (0.15), F/B (0.11) and O/P (0.07). The kinship coefficient between individuals in the Deli x Nigerian family ranged from 0.35 to 0.62 with both mean and median at 0.48 (Additional file 1: Figure S2).

### GS accuracy for SSR-based models

On average, the trait with the highest accuracy was F/B (0.28), followed by S/F (0.25), K/F (0.19) and O/DM (0.19), O/P (0.18) and M/F (0.18). Averaging across all traits, machine learning methods out-performed all other methods, albeit only slightly. SVM having the highest accuracy for F/B (0.30), S/F (0.30) and O/P (0.28), and RF having the highest accuracy for K/F (0.24), M/F (0.34) and O/DM (0.28). However, SVM also has the lowest accuracy for K/F (0.14), RF has the lowest accuracy for F/B (0.19) and O/P (0.14) (Table 1).

**Table 1:** Mean accuracy of traits based on different SSR-based GS methods

GS Method	F/B	S/F	K/F	M/F	O/P	O/DM	Mean
RR-BLUP	0.28	0.25	0.17	0.14	0.17	0.14	0.19
BA	0.29	0.24	0.19	0.15	0.17	0.17	0.20
BB	0.29	0.24	0.21	0.14	0.16	0.16	0.20
BC	0.29	0.23	0.20	0.14	0.18	0.18	0.20
BL	0.28	0.24	0.17	0.14	0.17	0.16	0.19
BRR	0.29	0.23	0.18	0.13	0.18	0.19	0.20
SVM	0.30	0.30	0.14	0.22	0.28	0.22	0.24
RF	0.19	0.25	0.24	0.34	0.14	0.28	0.24
Mean	0.28	0.25	0.19	0.18	0.18	0.19	

BL – Bayes Lasso, BRR – Bayes Ridge Regression, BA – Bayes A, BB – Bayes B, BC – Bayes Cπ, SVM – support vector machine, RF – random forest

### GS accuracy for SNP-based models

**Fig. 3:**
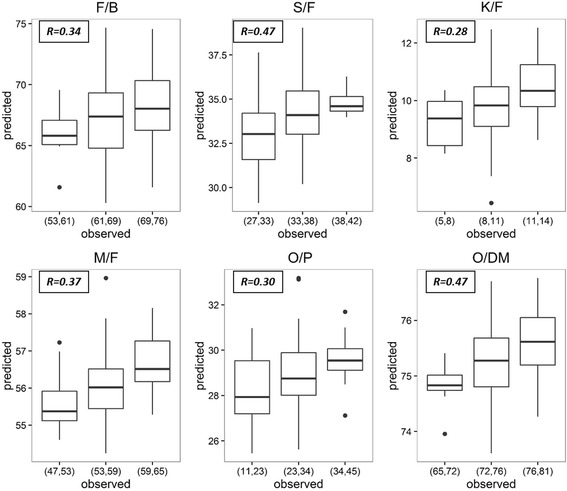
Regression boxplot illustrating predicted trait values vs. observed trait values for F/B, S/F, K/F, M/F, O/P and O/DM, selected by best GS method for each trait. The observed trait values were split into three classes. The prediction accuracy was written on the top left corner for each plot

Accuracy of the GS model was improved drastically for all traits when SNPs were used (Table 2 & Fig. 3). The improvement was the largest for S/F, M/F and O/DM. Averaging across methods, the trait with the highest accuracy was O/DM (0.43), S/F (0.39), followed by F/B (0.31) and by M/F (0.30). Averaging across traits, similar to SSRs, the performance of all methods were almost equal, with machine learning methods having a marginal advantage (0.32) over other methods (0.31). For individual traits, machine learning methods have the highest accuracy for all traits besides F/B, with SVM having the highest accuracy for S/F (0.47) and K/F (0.28), and RF having the highest accuracy for M/F (0.37), O/P (0.30) and O/DM (0.47). However, SVM also has the lowest accuracy M/F (0.26), RF has the lowest accuracy for F/B (0.24) and S/F (0.23). Residual analysis carried out for RR-BLUP and Bayesian methods showed that the points in a residual plot were randomly dispersed around the horizontal axis, and the points in QQ plot were almost in a straight line for all traits (Additional file 1: Figure S3 & Figure S4). Therefore, the underlying mixed linear model used was suitable.

**Table 2:** Mean accuracy of traits based on different SNP-based GS methods

GS Method	F/B	S/F	K/F	M/F	O/P	O/DM	Mean
RR-BLUP	0.31	0.40	0.18	0.30	0.21	0.42	0.30
BA	0.33	0.40	0.20	0.30	0.20	0.43	0.31
BB	0.34	0.40	0.19	0.30	0.20	0.43	0.31
BC	0.33	0.40	0.20	0.29	0.20	0.42	0.31
BL	0.30	0.39	0.19	0.28	0.18	0.42	0.29
BRR	0.32	0.40	0.20	0.29	0.20	0.42	0.30
SVM	0.32	0.47	0.28	0.26	0.27	0.39	0.33
RF	0.24	0.23	0.27	0.37	0.30	0.47	0.31
Mean	0.31	0.39	0.21	0.30	0.22	0.43	

BL – Bayes Lasso, BRR – Bayes Ridge Regression, BA – Bayes A, BB – Bayes B, BC – Bayes Cπ, SVM – support vector machine, RF – random forest

## Discussion

Perennial crops, including oil palm, have long life and crop cycles, require lengthy periods to reach maturity. Thus, important agricultural traits, such as oil yield can only be evaluated after years of field planting. With the planted materials remaining in production over long time frames, small differences in yield have a large impact. The introduction of genomic selection will enable early selection of the best performing parents without field evaluation, eventually expediting genetic gains in perennial crops. For oil palm, early trait prediction of the best performing Deli x Nigerian *dura* allows breeders to prioritize the best *dura* candidates to be progeny tested with the commercial AVROS pollen donors. The traits evaluated ranged from simple quantitative traits, which are controlled by fewer genes, including S/F [15] and O/DM [37], to the more complex quantitative trait controlled by more genes, O/P. From the statistical analysis of the traits in this study, M/F was inversely correlated with S/F and K/F, which was expected because the thicker the shell and kernel, the thinner the mesocarp, and vice versa. With crude palm oil accumulating in the mesocarp, fruits with thicker mesocarp are often preferred. As for the complex trait O/P, it was calculated from a few simple quantitative traits. Therefore positive correlations between O/P with M/F and O/DM were observed. Unlike annual crops, traits measured for a single year are not useful because oil palm yields vary greatly from year to year [38]. In comparison, The four-year mean (4th – 8th years of planting) for oil palm traits, such as FFB, provides higher heritability [39] and also highly correlates to later yields (11th – 16th years of planting) [40]. Consequently, the four-year mean was used in this study for all traits.

The conventional MAS only requires a few major QTLs for breeding prediction, but the genetic effects of individual QTL can be small for complex traits, with a limited amount of the trait variation explained [41]. In such conditions, the genetic gains will remain minimal or insignificant in the breeding program. The variations of complex traits can be further improved by consolidating the effects of all genes or chromosomal/genomic segments to estimate total breeding value, which is essentially the GS approach [31]. Conventionally, the thick-shelled *dura* as maternal parents are field planted in small number. Yet even with a small population, Cros [10] demonstrated that SSRs can be used in the GS modeling for *dura* palms, thereby enabling genomic predictions to be evaluated as a method to improve desired traits.

Various methods developed for GS make different assumptions regarding the markers. As compared to these methods, the absence of such assumptions might allow for machine learning to model the traits better. Among the methods assessed, even though the difference in performance was small, machine learning models did have the highest accuracy, followed by Bayesian methods and RR-BLUP. This slight increase in accuracy when using machine learning methods, was however, at the cost of longer computing times compared to Bayesian methods, which in turn took longer than RR-BLUP. In addition, machine learning methods gave inconsistent results among traits in this study. Different parameters are required for different traits using machine learning methods to maximize prediction accuracies. Determination of these parameters is again a computationally intensive and time consuming process. When the number of variables is large, such as in the case of high density SNPs in predictive modeling, parameters optimization is a challenging process. Nonetheless, given sufficient optimization, machine learning has the potential to outperform other methods.

Overall, the accuracy using SSRs alone was low for most of the traits, which is not in agreement to what was reported by Cros [10]. This is probably due to the lower kinship coefficient within the Deli x Nigerian family of 0.48 (current study), compared to 0.58 in the population used by Cros [10]. In addition, observed trait data was used in this study, instead of deregressed estimated breeding value derived from progeny testing. Hence, the observed trait data probably increased the residual effects due to environmental effect, thus reducing the accuracy. To be accurate, GS requires every QTL in the genome to be represented by at least one marker [42]. Even though it is possible to have genome-wide representation of SSRs, this is usually not done in practice due to the large amount of workload and the high cost required for genotyping. Therefore, usually it is insufficient for SSRs to cover all possible loci affecting a complex trait, and thus for use in GS of traits at underrepresented loci. In soybean, the accuracy of GS using low density markers has been reported to reach 0.69 [43]. However, the high accuracy might due to the use of trait-associated markers. In this case, the assayed SSRs in this study were randomly mined from the genome, thus no *a priori *association was known. As compared to SSRs, the introduction of high-throughput SNP genotyping has made whole-genomic genotyping possible at a low cost.

When genome-wide SNPs were introduced into the GS models, accuracy for all traits improved, with the greatest improvement for S/F, M/F and O/DM. This can be attributed to the fact that the OP200K array provides high genomic resolution [15]. With most of the QTL being represented by at least one SNP, most of the important genetic variations relative to the traits in study would have been captured. Representing SNP as marker effect, the model built from the genome-wide SNPs therefore better explained the observed trait variations. In addition to marker density, another important factor that affects accuracy is trait heritability. The genomic heritabilities calculated for the traits in this study were in the descending order of S/F, O/DM, M/F, K/F, F/B and lastly O/P. The fruit traits, including S/F, M/F, K/F and O/DM were reported to be highly heritable in an introgressed Deli *dura* x Nigerian *dura* population, while F/B and O/P were reported to have comparatively lower heritability [44]. Therefore, the calculated genomic heritabilities agreed with the literature rather well. Among the highly heritable traits, K/F had the lowest genomic heritability, which coincided with it having the lowest accuracy among the highly heritable traits. The low heritability and accuracy were probably due to the low phenotypic variation of K/F in the assayed family. Another important factor is the training population size. Even though further improvement in accuracy can easily be achieved through the increment in the training population size [45], the *dura* maternal population size is usually small in breeding programs, as they are often accommodated in commercial estates. In these estates, *tenera* palms (the hybrids used for oil production) are predominantly planted, together with a small number of mother (*dura*) palms, which are used solely for seed production for new planting materials. The small training population size in this study therefore reflects the reality of *dura* mother palm breeding. Even with this condition, we have found the application of GS in *dura* breeding programs to be promising.

## Conclusion

For outcrossing perennial crops such as oil palm, the conventional breeding selection of mother palms for progeny testing is partially random and is phenotype-dependent. The ability to select for the best mother palms will expedite the production of high-yielding progenies as future commercial planting materials. Despite having a small training population, which is a usual case for mother palms, GS using SSRs has reasonable accuracy, which can be much improved by using dense SNPs that afford better coverage of the genome and hence the desirable QTL. From our results, the differences in accuracies between the methods evaluated for SSRs and SNPs were small. In general machine learning methods slightly outperformed the other methods, and with sufficient parameter optimization machine learning could become a powerful tool to support selection of optimal materials for breeding programs of oil palm and other perennial crops.

## Additional file

Additional file 1: Figure S1. Estimation of optimal MCMC iterations required for Bayes A - S/F and O/P. **Figure S2.** Distribution of kinship coefficients for 112 Deli x Nigerian individuals used in this study. **Figure S3.** Residual plots for different prediction models for all traits. **Figure S4.** QQ plots for different prediction models for all traits (PDF 640 kb)

## Abbreviations

F/B: Fruit-to-bunch
FFB: Fresh fruit bunch
GS: Genomic selection
GWAS: Genome-wide association study
K/F: Kernel-to-fruit
M/F: Mesocarp-to-fruit
MAS: Marker assisted Selection
O/B: Oil-to-bunch
O/DM: Oil-to-dry mesocarp
O/P: Oil per palm
QTL: Quantitative trait loci
RF: Random forest
S/F: Shell-to-fruit
SNPs: Single nucleotide polymorphisms
SSRs: Simple sequence repeats
SVM: Support vector machine

## References

[1] Hayes BJ, Bowman PJ, Chamberlain AJ, Goddard ME (2009). Invited review: genomic selection in dairy cattle: progress and challenges. J Dairy Sci.

[2] Boichard D, Ducrocq V, Croiseau P, Fritz S (2016). Genomic selection in domestic animals: principles, applications and perspectives. C R Biol.

[3] Crossa J, Perez P, Hickey J, Burgueno J, Ornella L, Ceron-Rojas J, Zhang X, Dreisigacker S, Babu R, Li Y (2014). Genomic prediction in CIMMYT maize and wheat breeding programs. Heredity.

[4] Spindel J, Begum H, Akdemir D, Virk P, Collard B, Redona E, Atlin G, Jannink JL, McCouch SR (2015). Correction: genomic selection and association mapping in Rice (Oryza Sativa): effect of trait genetic architecture, training population composition, marker number and statistical model on accuracy of Rice genomic selection in elite, tropical Rice breeding lines. PLoS Genet.

[5] Kainer D, Lanfear R, Foley WJ, Kulheim C (2015). Genomic approaches to selection in outcrossing perennials: focus on essential oil crops. Theor Appl Genet.

[6] Mielke T. Oil world annual 2008. In: ISTA Mielke. Hamburg: ISTA Mielke; 2008.

[7] Hardon JJ (1970). Inbreeding in populations of the oil palm (Elaeis Guineensis Jacq.) and its effects on selection. Oleagineux.

[8] Oboh B, Fakorede M (1989). Optimum time for yield evaluation and selection in the oil palm. Oleagineux.

[9] Wong CK, Bernardo R (2008). Genomewide selection in oil palm: increasing selection gain per unit time and cost with small populations. Theor Appl Genet.

[10] Cros D, Denis M, Sanchez L, Cochard B, Flori A, Durand-Gasselin T, Nouy B, Omore A, Pomies V, Riou V (2015). Genomic selection prediction accuracy in a perennial crop: case study of oil palm (Elaeis Guineensis Jacq.). Theor Appl Genet.

[11] Marchal A, Legarra A, Tisné S, Carasco-Lacombe C, Manez A, Suryana E, Omoré A, Nouy B, Durand-Gasselin T, Sánchez L, et al. Multivariate genomic model improves analysis of oil palm (Elaeis Guineensis Jacq.) progeny tests. Mol Breed. 2016;36(2):1–13.

[12] Singh R, Low ET, Ooi LC, Ong-Abdullah M, Ting NC, Nagappan J, Nookiah R, Amiruddin MD, Rosli R, Manaf MA (2013). The oil palm SHELL gene controls oil yield and encodes a homologue of SEEDSTICK. Nature.

[13] Ooi LC, Low ET, Abdullah MO, Nookiah R, Ting NC, Nagappan J, Manaf MA, Chan KL, Halim MA, Azizi N (2016). Non-tenera contamination and the economic impact of SHELL genetic testing in the Malaysian independent oil palm industry. Front Plant Sci.

[14] Teh CK, Ong AL, Kwong QB, Apparow S, Chew FT, Mayes S, Mohamed M, Appleton D, Kulaveerasingam H (2016). Genome-wide association study identifies three key loci for high mesocarp oil content in perennial crop oil palm. Sci Rep.

[15] Kwong QB, Teh CK, Ong AL, Heng HY, Lee HL, Mohamed M, Low JZ, Sukganah A, Chew FT, Mayes S (2016). Development and validation of a high density SNP genotyping Array for African oil palm. Mol Plant.

[16] Singh R, Ong-Abdullah M, Low ET, Manaf MA, Rosli R, Nookiah R, Ooi LC, Ooi SE, Chan KL, Halim MA (2013). Oil palm genome sequence reveals divergence of interfertile species in old and new worlds. Nature.

[17] Kwong QB, Ong AL, Teh CK, Chew FT, Tammi M, Mayes S, Kulaveerasingam H, Yeoh SH, Harikrishna JA, Appleton DR (2017). Genomic selection in commercial perennial crops: applicability and improvement in oil palm (Elaeis Guineensis Jacq.). Sci Rep.

[18] Teh CK, Praveena T, Sukganah A, Ong AL, Mayes S, Chew FT, Appleton D: Identification of QTL for agronomical traits using linkage analysis and validated by GWAS in oil palm In Press.

[19] Blaak G, Sparnaaij LD, Menendez T (1963). Methods of bunch analysis. Breeding and inheritance in the oil palm *(Elaeis guineensis Jacq) Part II.* Vol. 4: J.W. Afr. Ins. Oil palm res.

[20] Rao V, Soh AC, Corley RHV, Lee CH, Rajanaidu N, Tan YP, Chin CW, Lim KC, Tan ST, Lee TP (1983). A critical reexamination of the method of bunch analysis in oil palm breeding. Palm Oil Research Institute Malaysia Occ Paper.

[21] Corley RHV, Tinker PBH: Selection and breeding. In: The oil palm Blackwell. Oxford: Blackwell. 2003.

[22] Peterson BG, Carl P, Boudt K, Bennett K, Ulrich J, Zivot EL, M., Balkissoon K, Wuertz D: Econometric tools for performance and risk analysis. In*.*, 1.4.3541 edn. https://cran.r-project.org/web/packages/PerformanceAnalytics/; 2015. Accessed 21 Apr 2015.

[23] Blair MW, Hedetale V, McCouch SR (2002). Fluorescent-labeled microsatellite panels useful for detecting allelic diversity in cultivated rice ( Oryza Sativa L.). Theor Appl Genet.

[24] Schuelke M (2000). An economic method for the fluorescent labeling of PCR fragments. Nat Biotechnol.

[25] Liaw MW A (2002). Classification and regression by randomForest. R News.

[26] de Los Campos G, Sorensen D, Gianola D (2015). Genomic heritability: what is it?. PLoS Genet.

[27] Yang J, Benyamin B, McEvoy BP, Gordon S, Henders AK, Nyholt DR, Madden PA, Heath AC, Martin NG, Montgomery GW (2010). Common SNPs explain a large proportion of the heritability for human height. Nat Genet.

[28] Pew J, Muir PH, Wang J, Frasier TR (2015). Related: an R package for analysing pairwise relatedness from codominant molecular markers. Mol Ecol Resour.

[29] Li CC, Weeks DE, Chakravarti A (1993). Similarity of DNA fingerprints due to chance and relatedness. Hum Hered.

[30] Endelman JB (2011). Ridge regression and other kernels for genomic selection with R package rrBLUP. Plant Genome.

[31] Meuwissen TH, Hayes BJ, Goddard ME (2001). Prediction of total genetic value using genome-wide dense marker maps. Genetics.

[32] Habier D, Fernando RL, Kizilkaya K, Garrick DJ (2011). Extension of the bayesian alphabet for genomic selection. BMC bioinformatics.

[33] Legarra A, Robert-Granie C, Croiseau P, Guillaume F, Fritz S (2011). Improved Lasso for genomic selection. Genet Res.

[34] Perez P, De los Campos G (2014). Genome-wide regression and prediction with the BGLR statistical package. Genetics.

[35] Perez P, de Los Campos G, Crossa J, Gianola D (2010). Genomic-enabled prediction based on molecular markers and pedigree using the Bayesian linear regression package in R. Plant Genome.

[36] Hsu C-W, Chang C-C, Lin C-J: A Practical Guide to Support Vector Classification. In*.*; 2003.

[37] Teh C-K, Ong A-L, Kwong Q-B, Apparow S, Chew F-T, Mayes S, Mohamed M, Appleton D, Kulaveerasingam H (2016). Genome-wide association study identifies three key loci for high mesocarp oil content in perennial crop oil palm. Sci Rep.

[38] Corley RHV, Lee CH, Law IH, Cundall E. Field testing of oil palm clones. In: Oil Palm Conference 'Progress and Prospects'. Kuala Lumpur: Palm Oil Research Institute of Malaysia. 1988. p. 173–85.

[39] Okwuagwu CO, Tai GCC (1995). Estimation of variance components and heritability of bunch yield and yield components in the oil palm (Elaeis Guineensis Jacq.). Pl Breed.

[40] Blaak G (1965). Breeding and inheritance in the oil palm (Elaeis Guineensis Jacq.) part III. Yield selection and inheritance. J W Afr Inst Oil Palm Res.

[41] Bernardo R. Molecular markers and selection for complex traits in plants: learning from the last 20 years. Crop Sci. 2008;48:1649–64.

[42] Goddard ME, Hayes BJ (2007). Genomic selection. Journal of animal breeding and genetics = Zeitschrift fur Tierzuchtung und Zuchtungsbiologie.

[43] Shu YJ, DS Y, Wang D, Bai X, Zhu YM, Guo CH (2013). Genomic selection of seed weight based on low-density SCAR markers in soybean. Genet Mol Res.

[44] Noh A, Rafii MY, Mohd Din A, Kushairi A, Norziha A, Rajanaidu N, Latif MA, Malek MA (2014). Variability and performance evaluation of introgressed Nigerian dura x deli dura oil palm progenies. Genet Mol Res.

[45] Lorenz AJ, Chao S, Asoro FG, Heffner EL, Hayashi T, Iwata H, Smith KP, Sorrells ME, Jannink JL. Genomic Selection in Plant Breeding:: Knowledge and Prospects. In: Sparks DL, editor. Advances in Agronomy, vol. 110: C edn. New York: Elsevier Inc. 2011.

