# Supplementary material for: Evaluation of methods and marker Systems in Genomic Selection of oil palm (Elaeis guineensis Jacq.)
Source: BMC Genet. 2017 Dec 11;18:107. doi: 10.1186/s12863-017-0576-5 (PMC5725918; doi:10.1186/s12863-017-0576-5)

Supplementary information

Evaluation of Methods and Marker Systems in Genomic Selection of Oil Palm (*Elaeis guineensis* Jacq.)

Qi Bin Kwong*[1,2] , Chee Keng Teh[1], Ai Ling Ong[1], Fook Tim Chew[3] Sean Mayes[4], Harikrishna Kulaveerasingam[1] ,Martti Tammi[1] ,Suat Hui Yeoh[2], David Ross Appleton[1], Jennifer Ann Harikrishna*[2,5]

[1] Biotechnology & Breeding Department, Sime Darby Plantation R&D Centre, Selangor, 43400, Malaysia

[2] Institute of Biological Sciences, University Malaya, 50603, Kuala Lumpur

[3] Department of Biological Sciences, National University of Singapore, 117543, Singapore

[4] School of Biosciences, University of Nottingham, Sutton Bonington Campus, Nr Loughborough, LE12 5RD, UK

[5]Centre of Research in Biotechnology for Agriculture (CEBAR), University of Malaya, 50603, Kuala Lumpur, Malaysia.

* Corresponding author:

Qi Bin Kwong (kwong.qi.bin.@simedarby.com)

Jennifer Ann Harikrishna (jennihari@um.edu.my)

Supplementary Figure 1. Estimation of optimal MCMC iterations required for Bayes A method – S/F and O/P

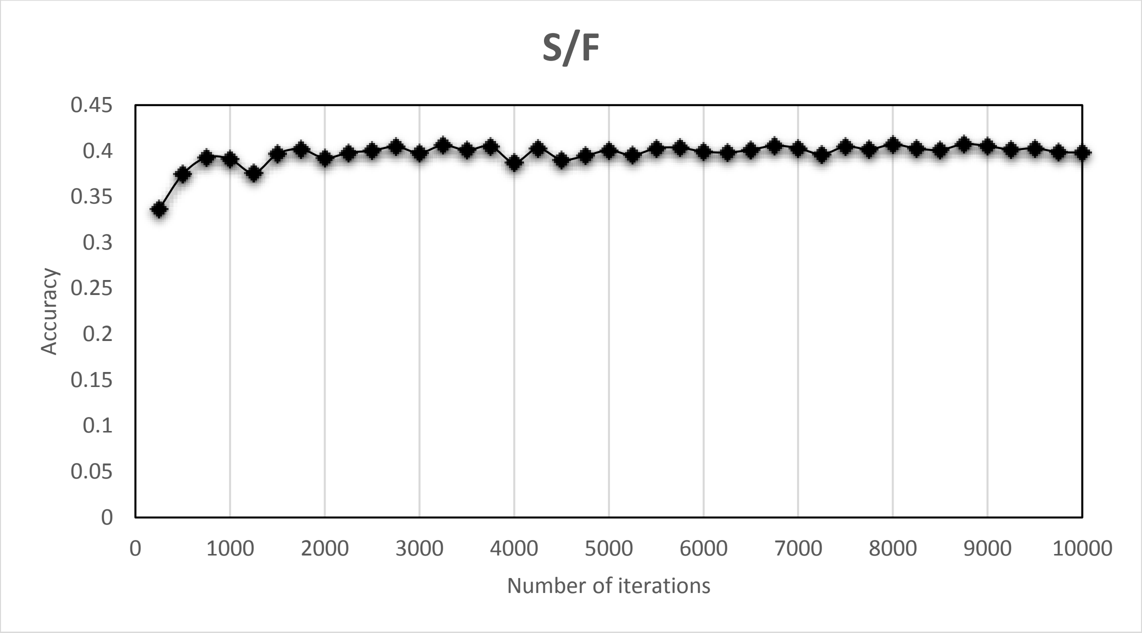

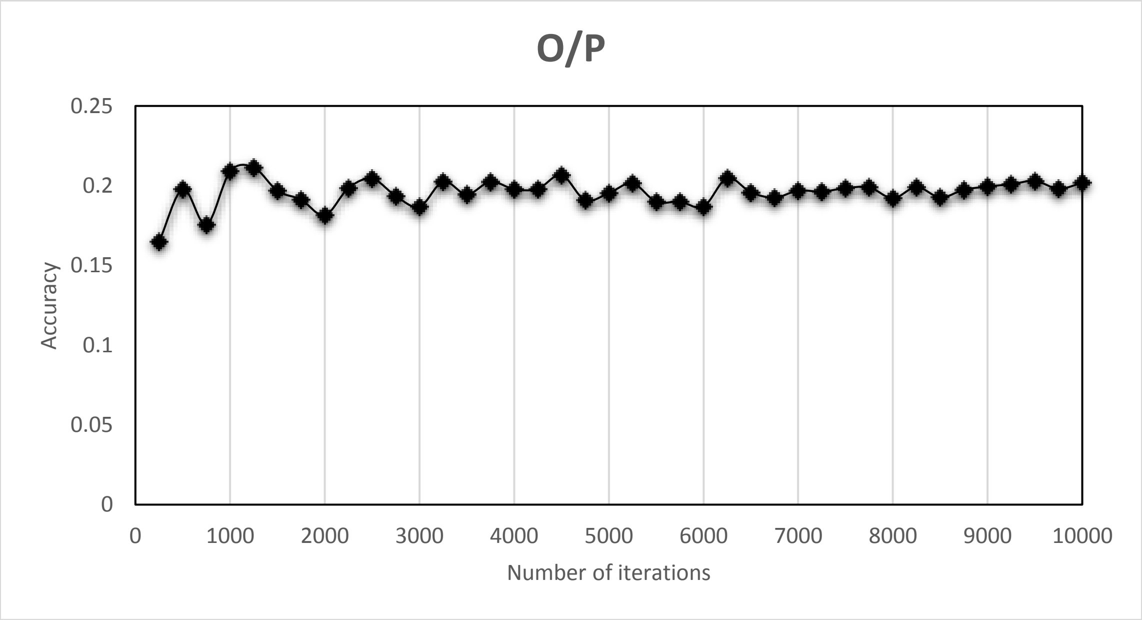

Supplementary Figure 2. Distribution of kinship coefficients for 112 Deli x Nigerian individuals used in this study

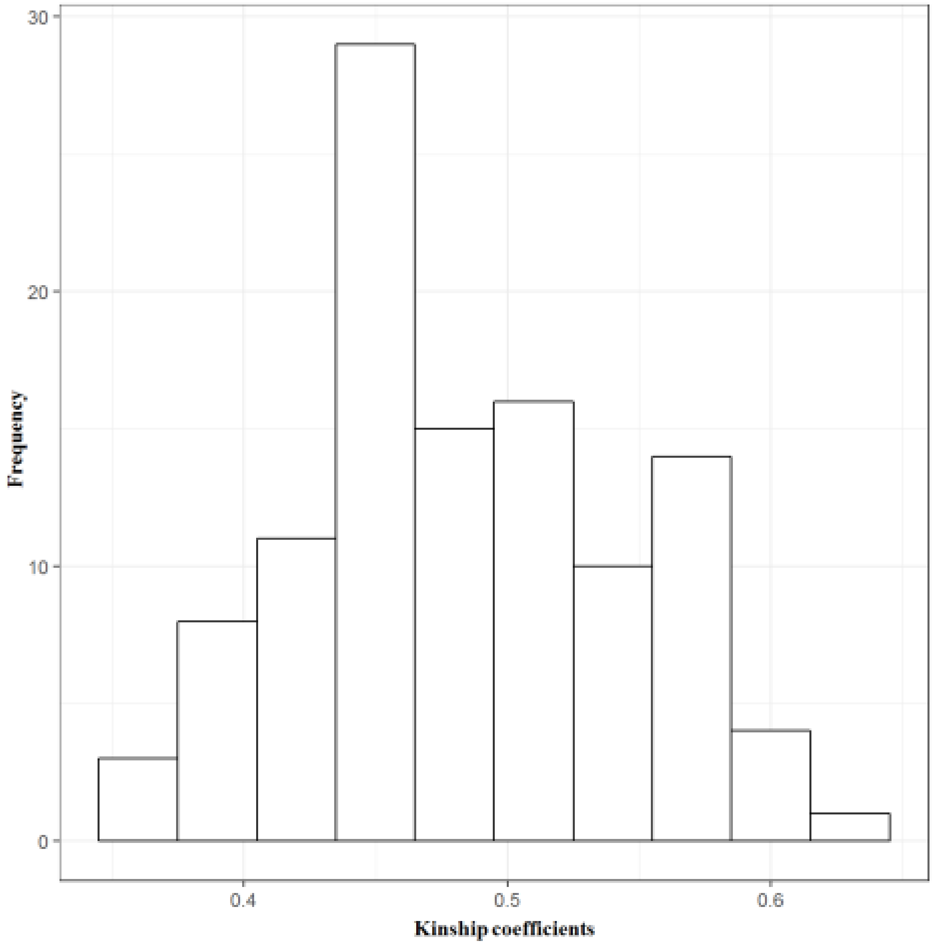

Supplementary Figure 3(a). Residual plots using RR-BLUP prediction models for all traits

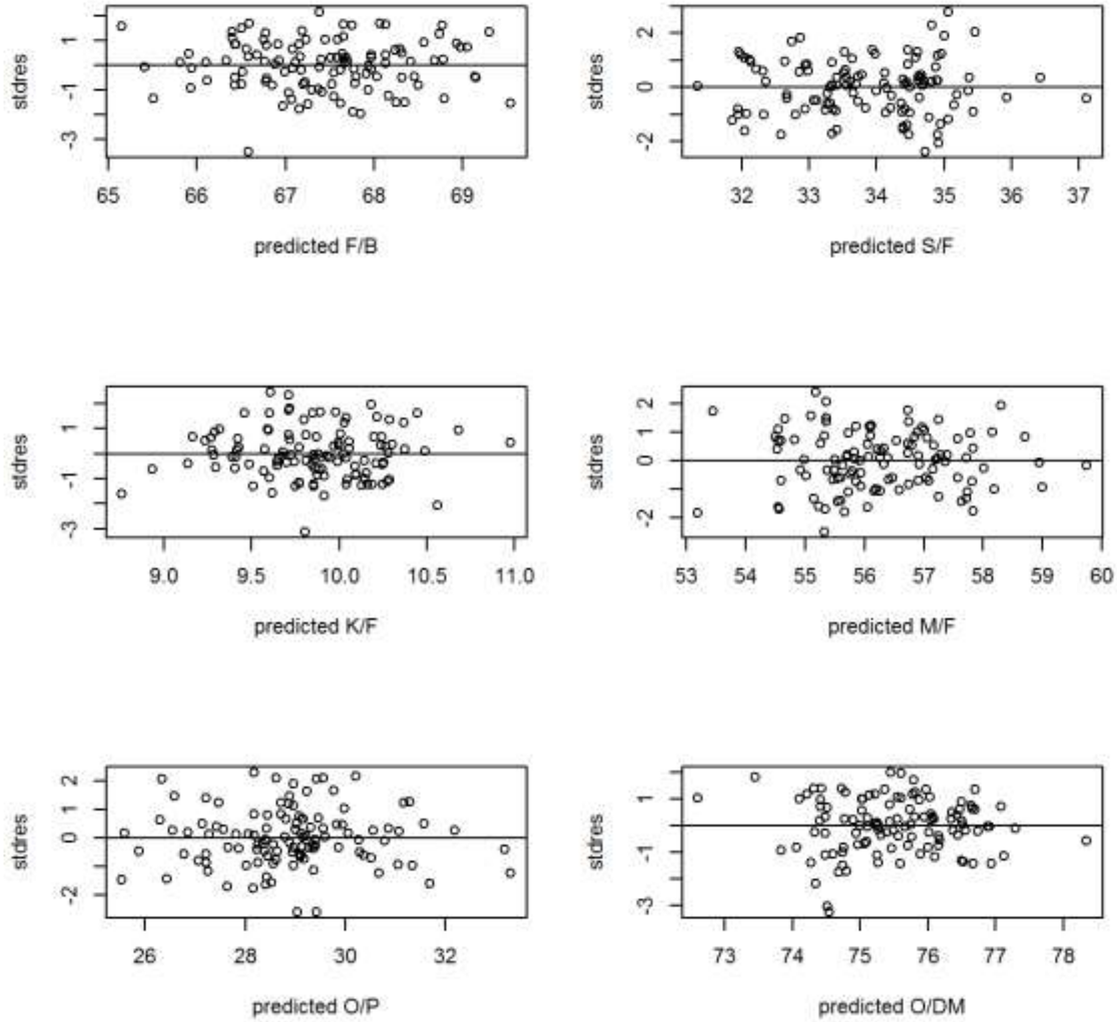

Supplementary Figure 3(b). Residual plots using BA prediction models for all traits

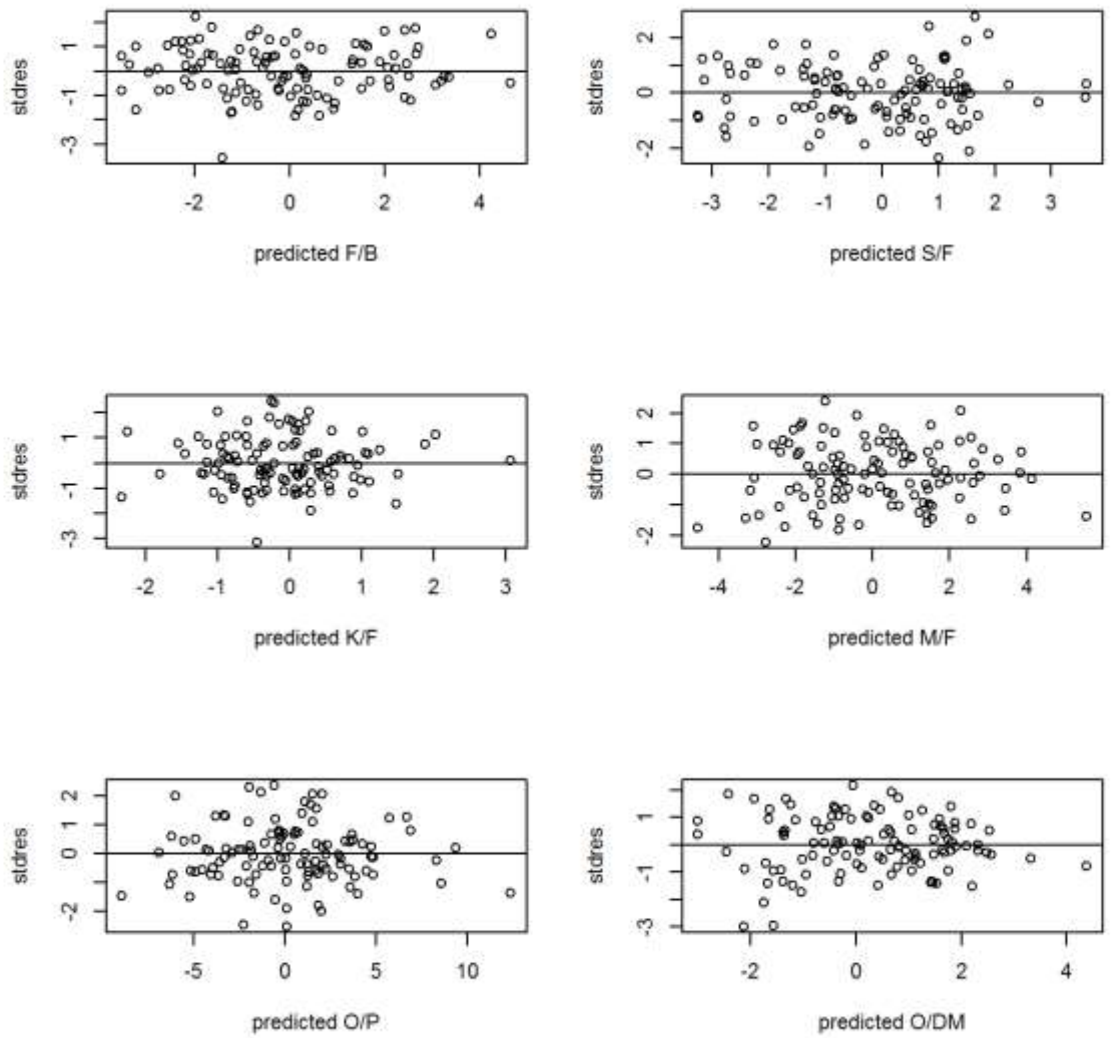

Supplementary Figure 3(c). Residual plots using BB prediction models for all traits

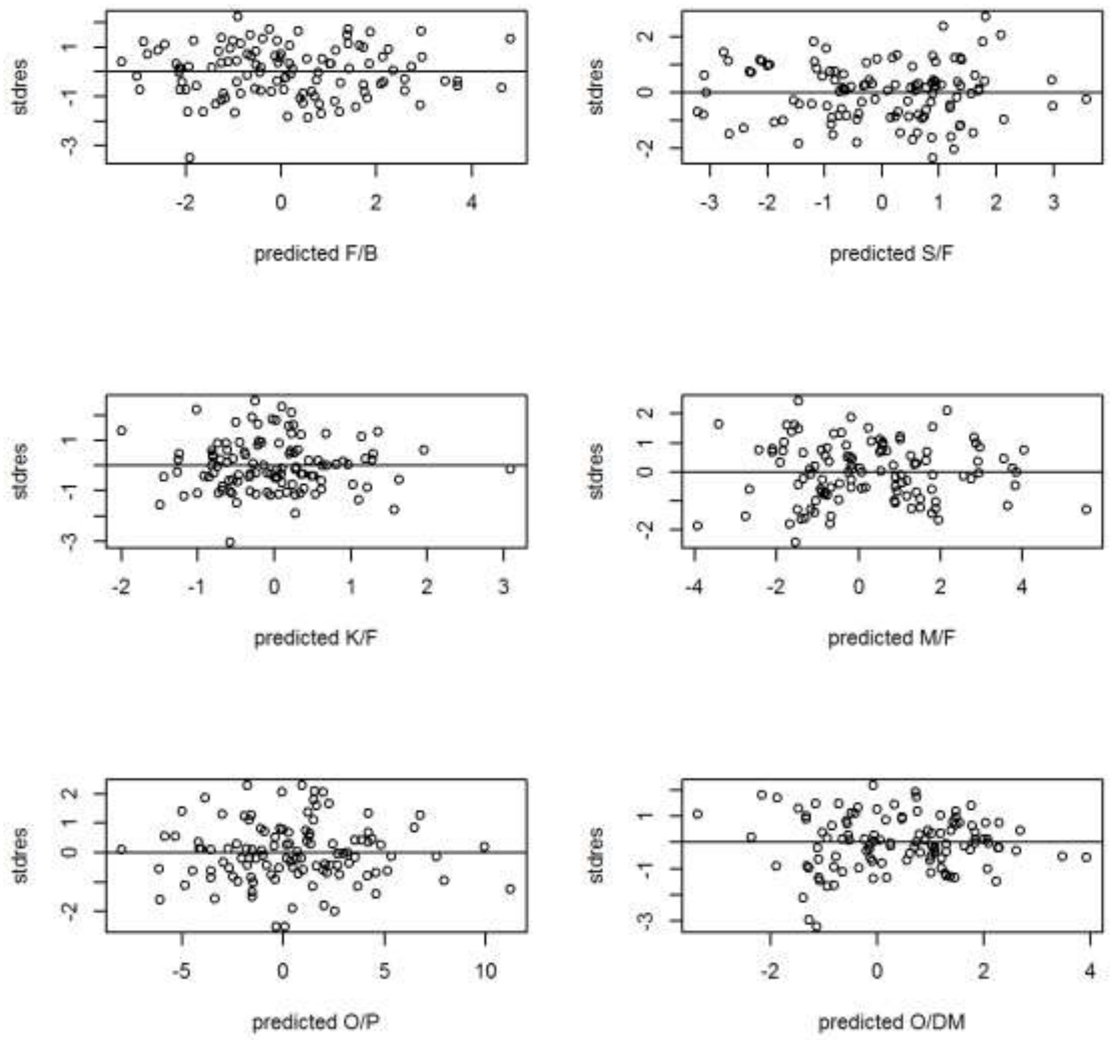

Supplementary Figure 3(d). Residual plots using BC prediction models for all traits

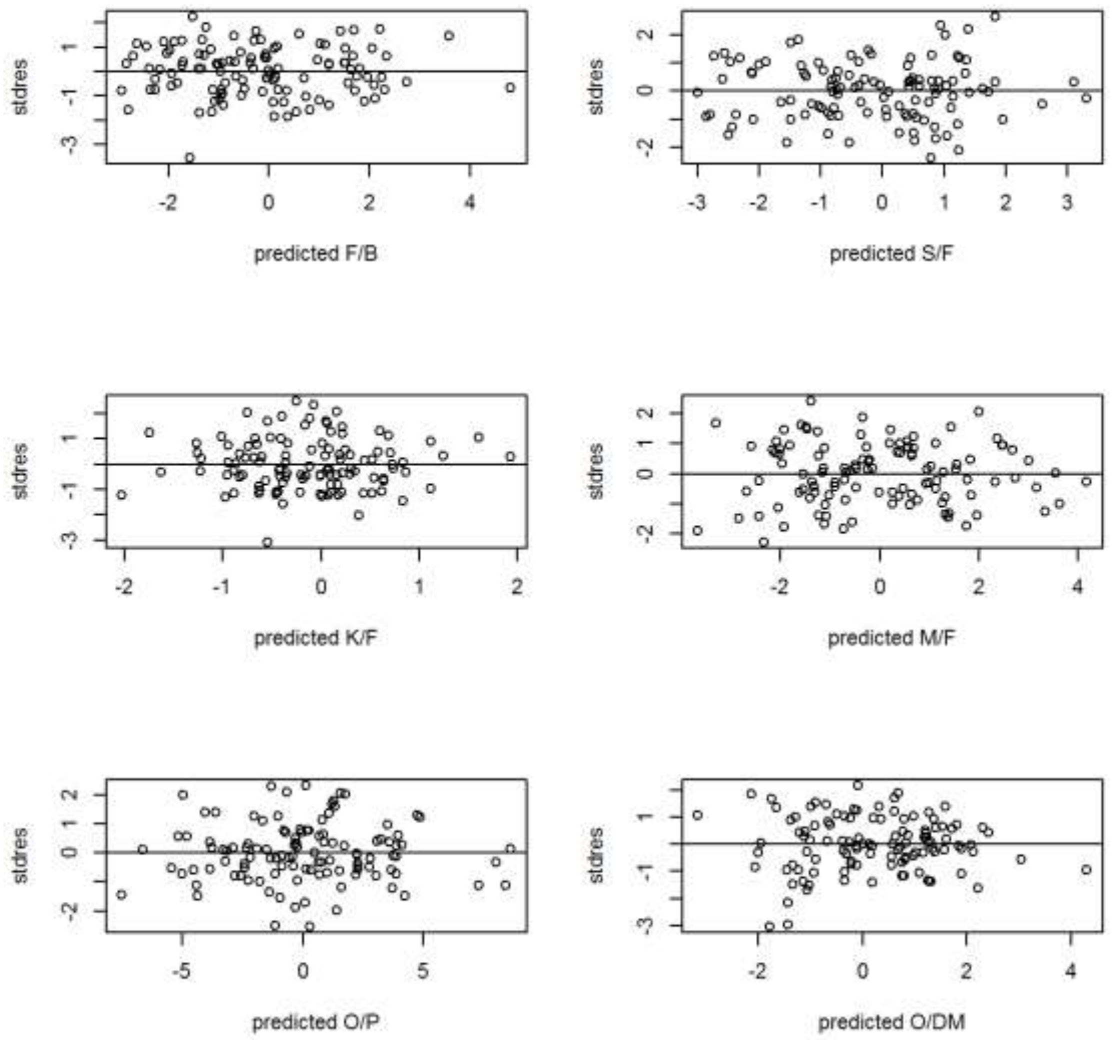

Supplementary Figure 3(e). Residual plots using BL prediction models for all traits

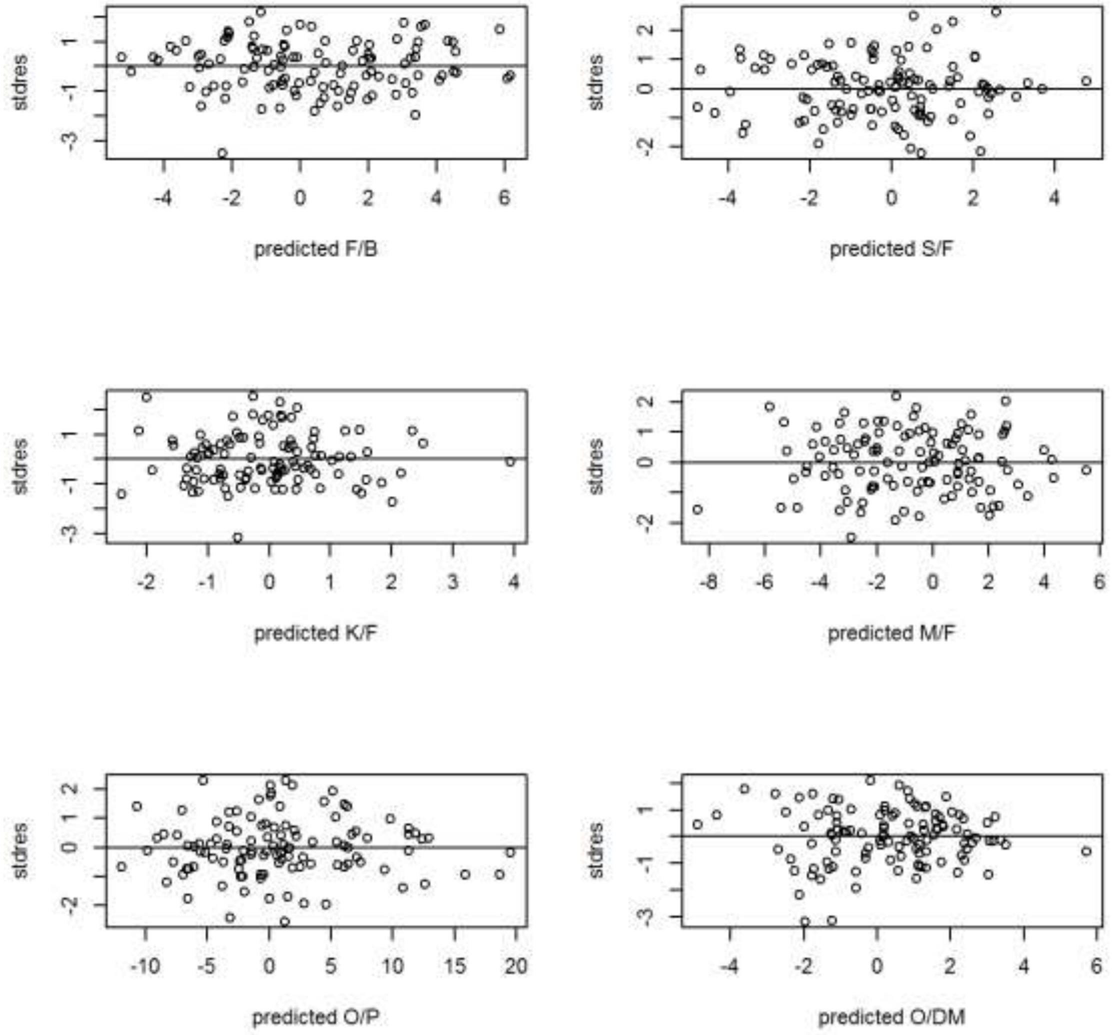

Supplementary Figure 3(f). Residual plots using BRR prediction models for all traits

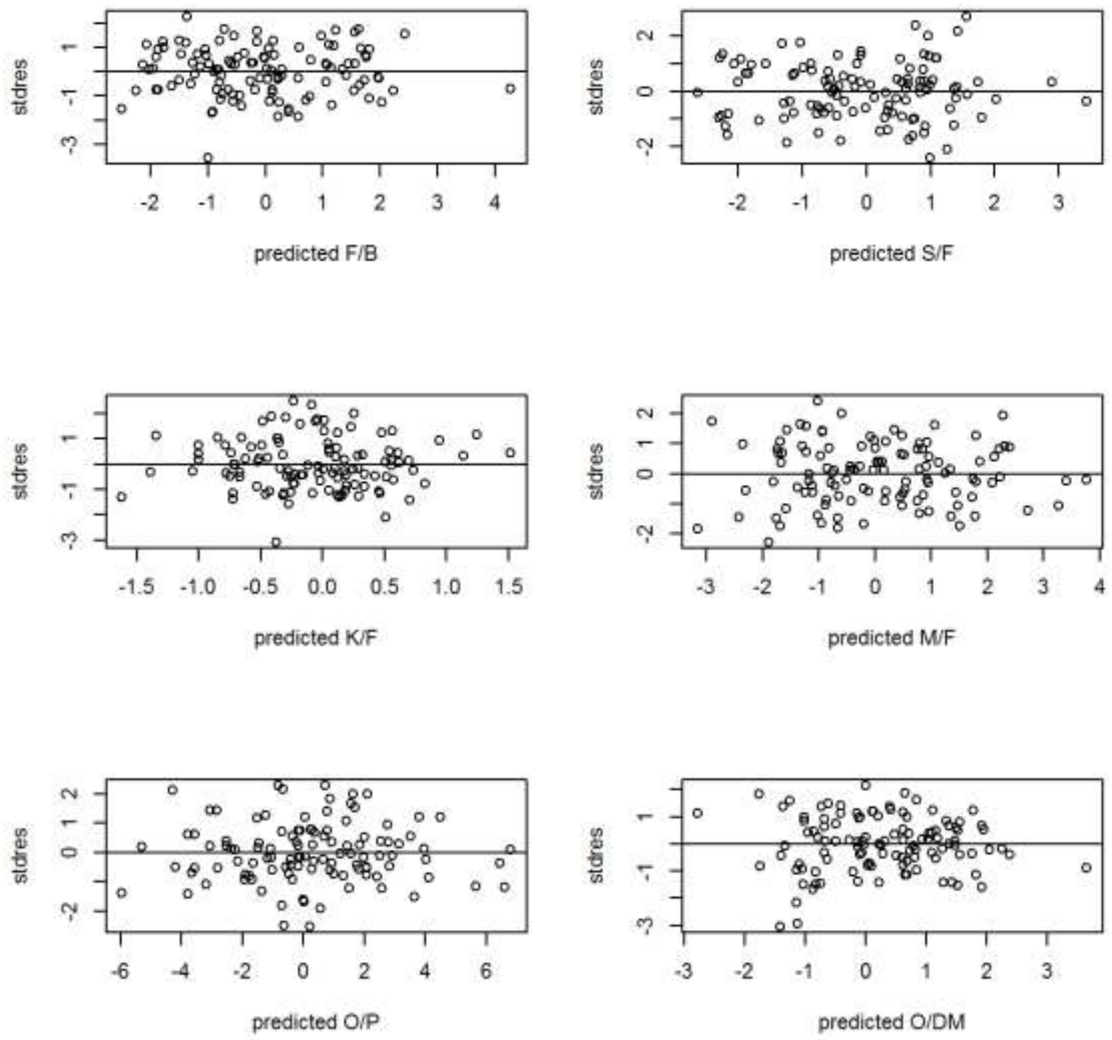

Supplementary Figure 4(a). QQ plots using RR-BLUP prediction models for all traits

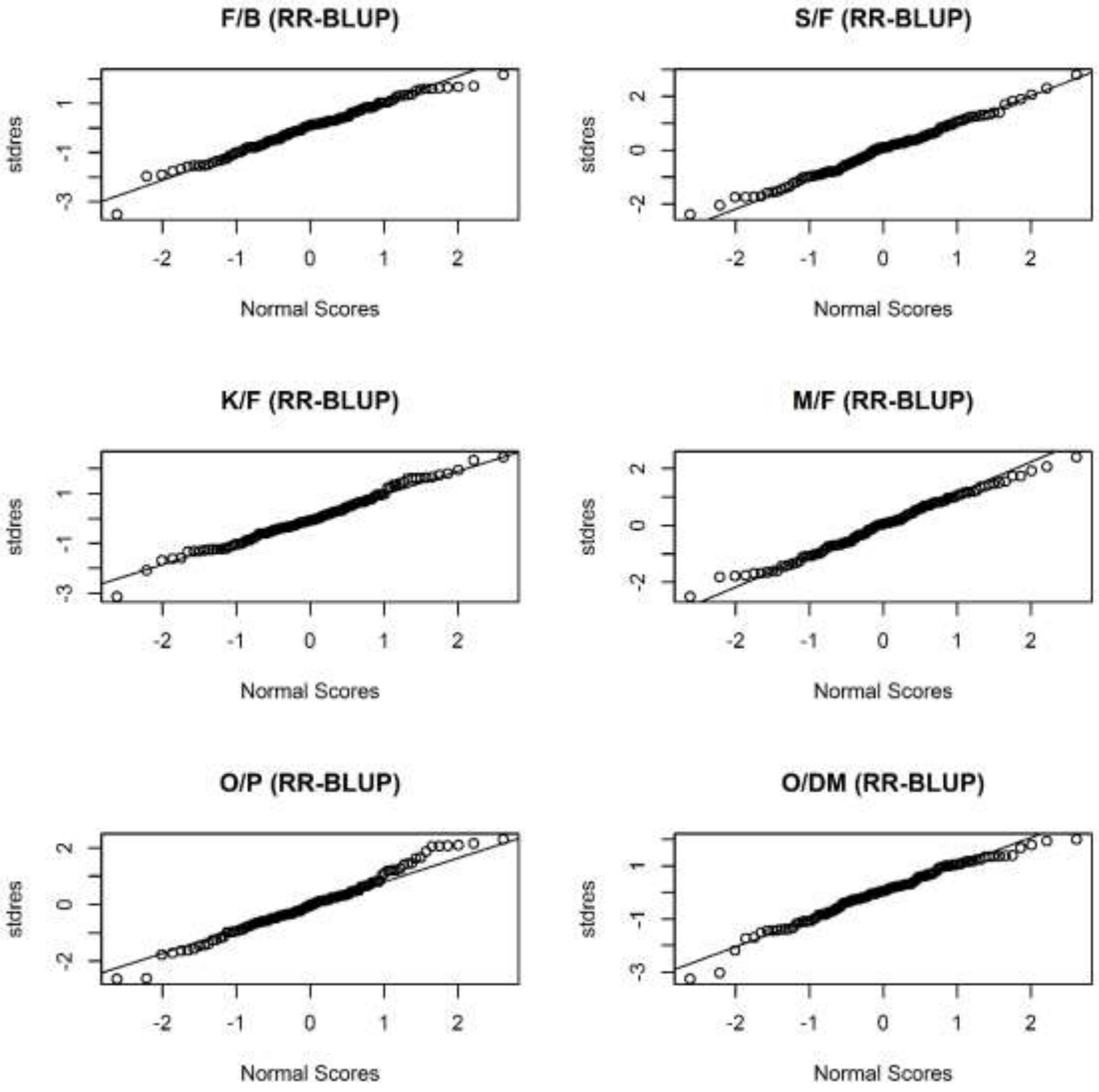

Supplementary Figure 4(b). QQ plots using BA prediction models for all traits

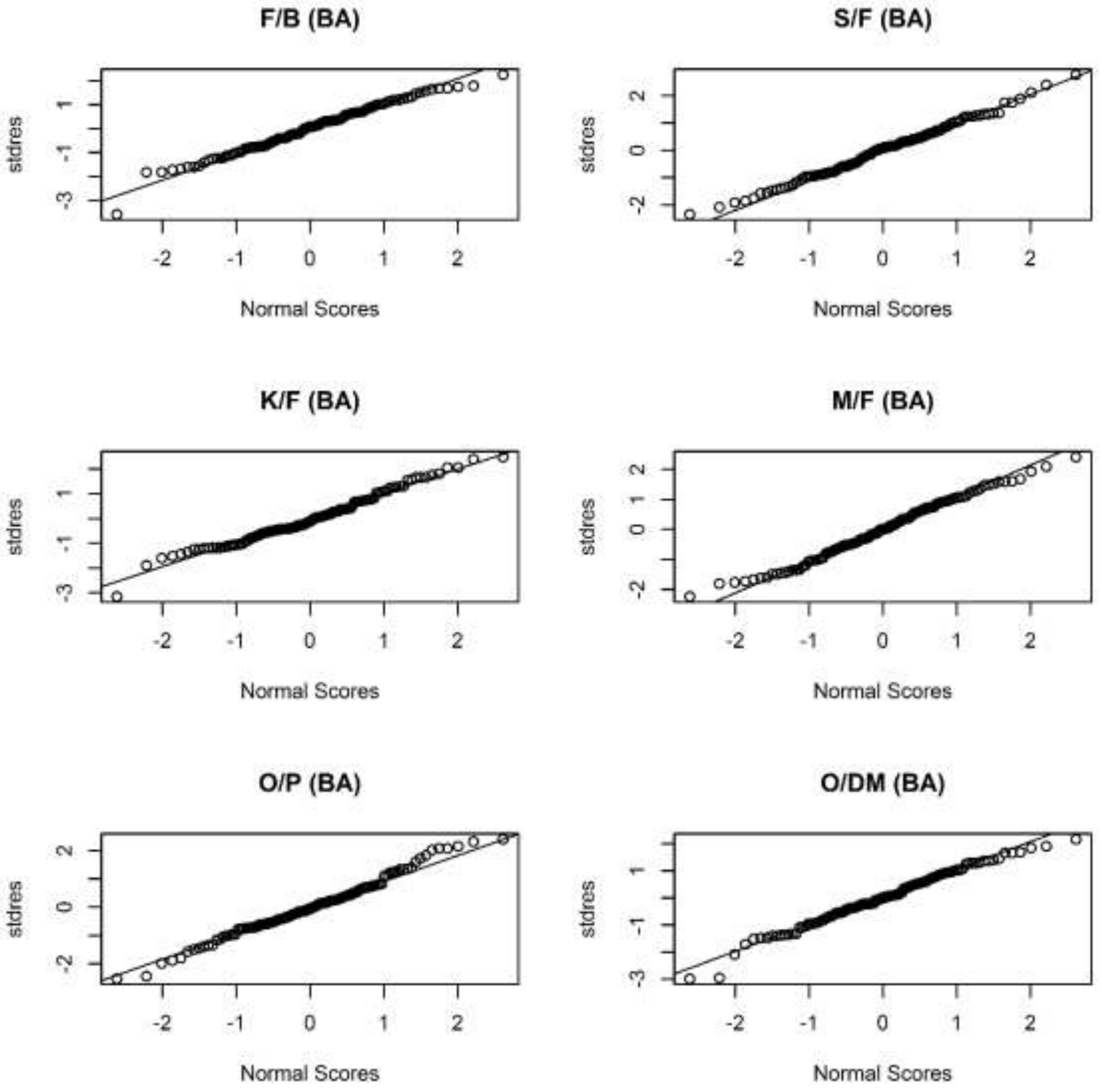

Supplementary Figure 4(c). QQ plots using BB prediction models for all traits

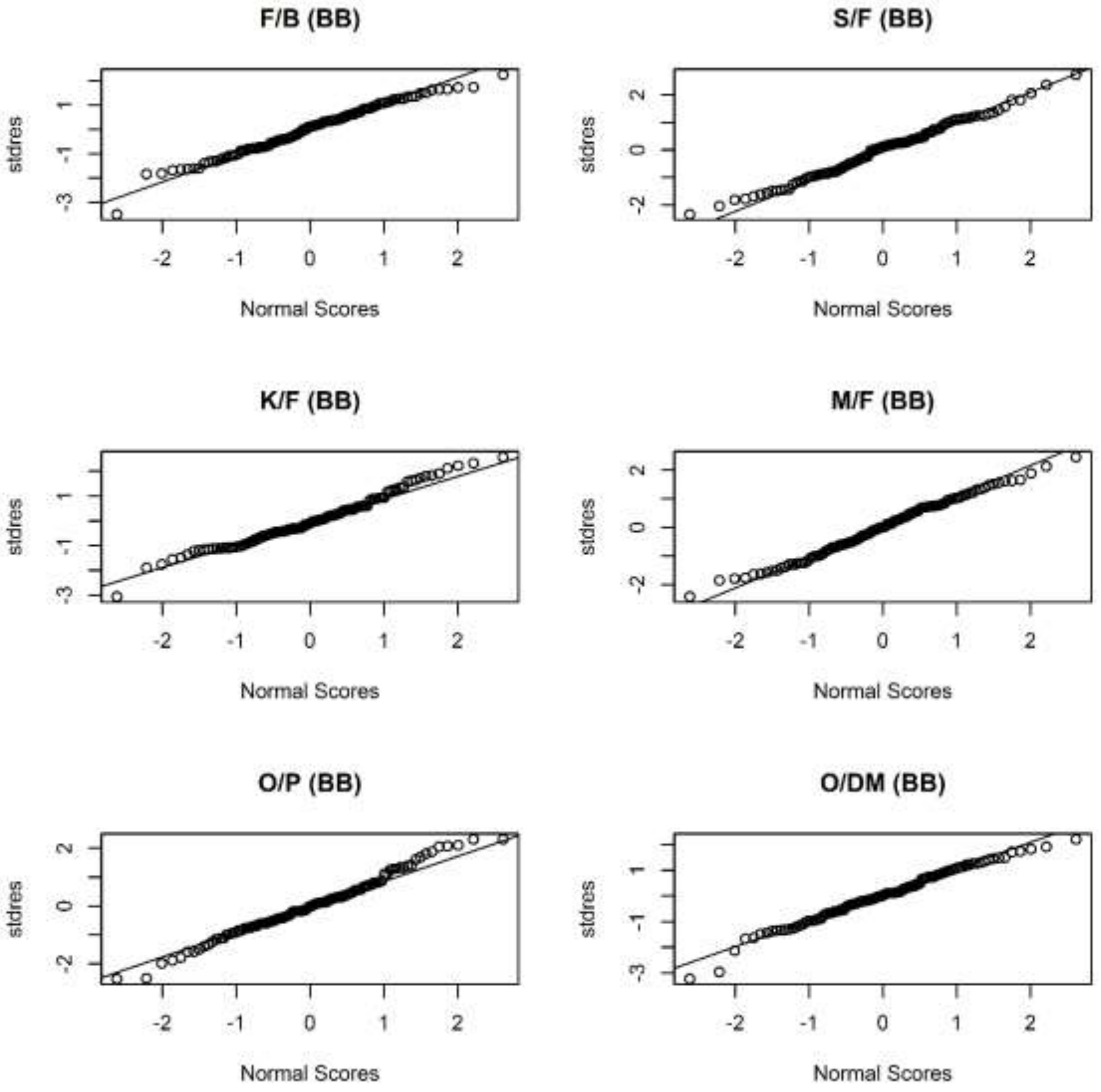

Supplementary Figure 4(d). QQ plots using BC prediction models for all traits

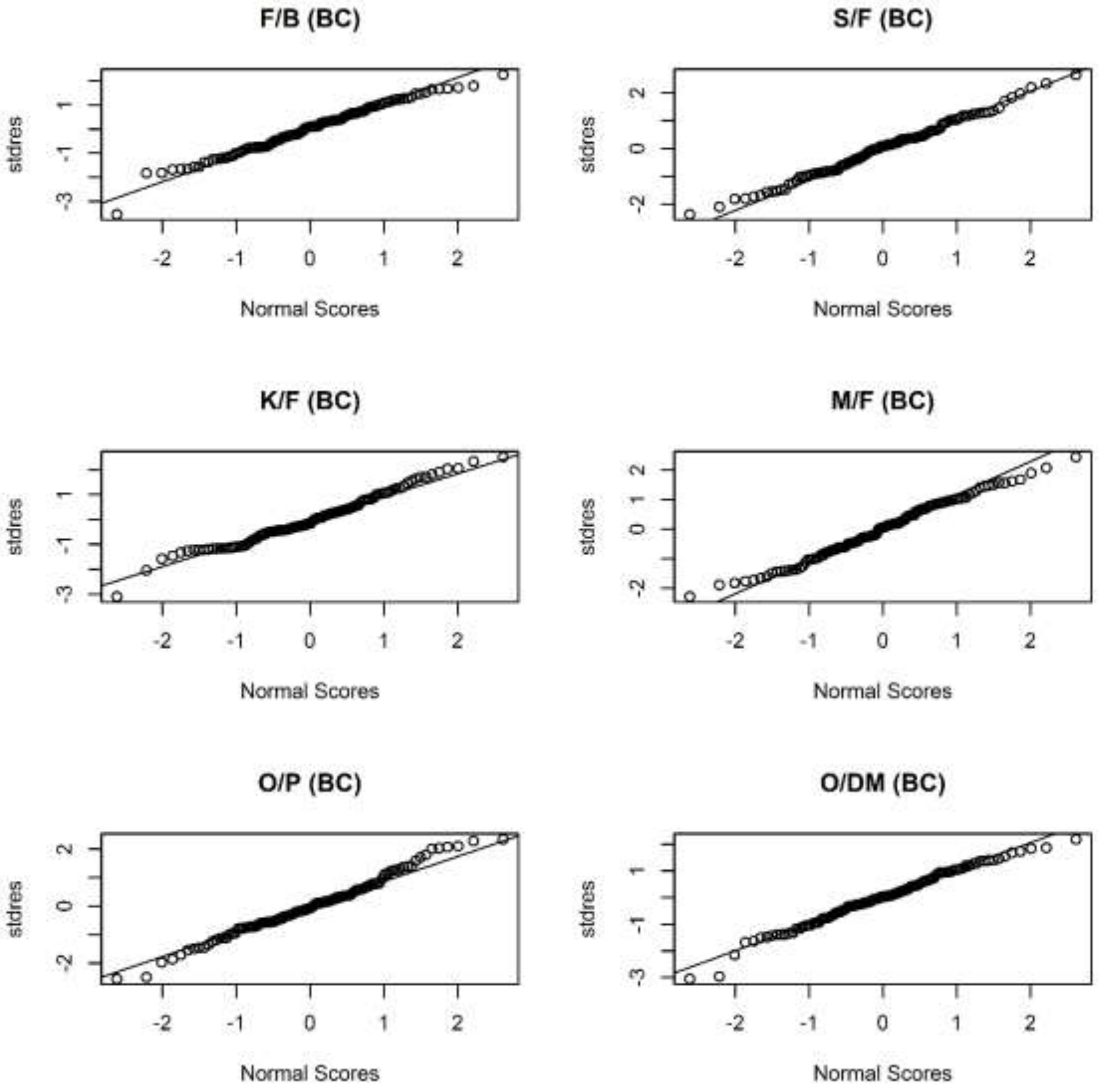

Supplementary Figure 4(e). QQ plots using BL prediction models for all traits

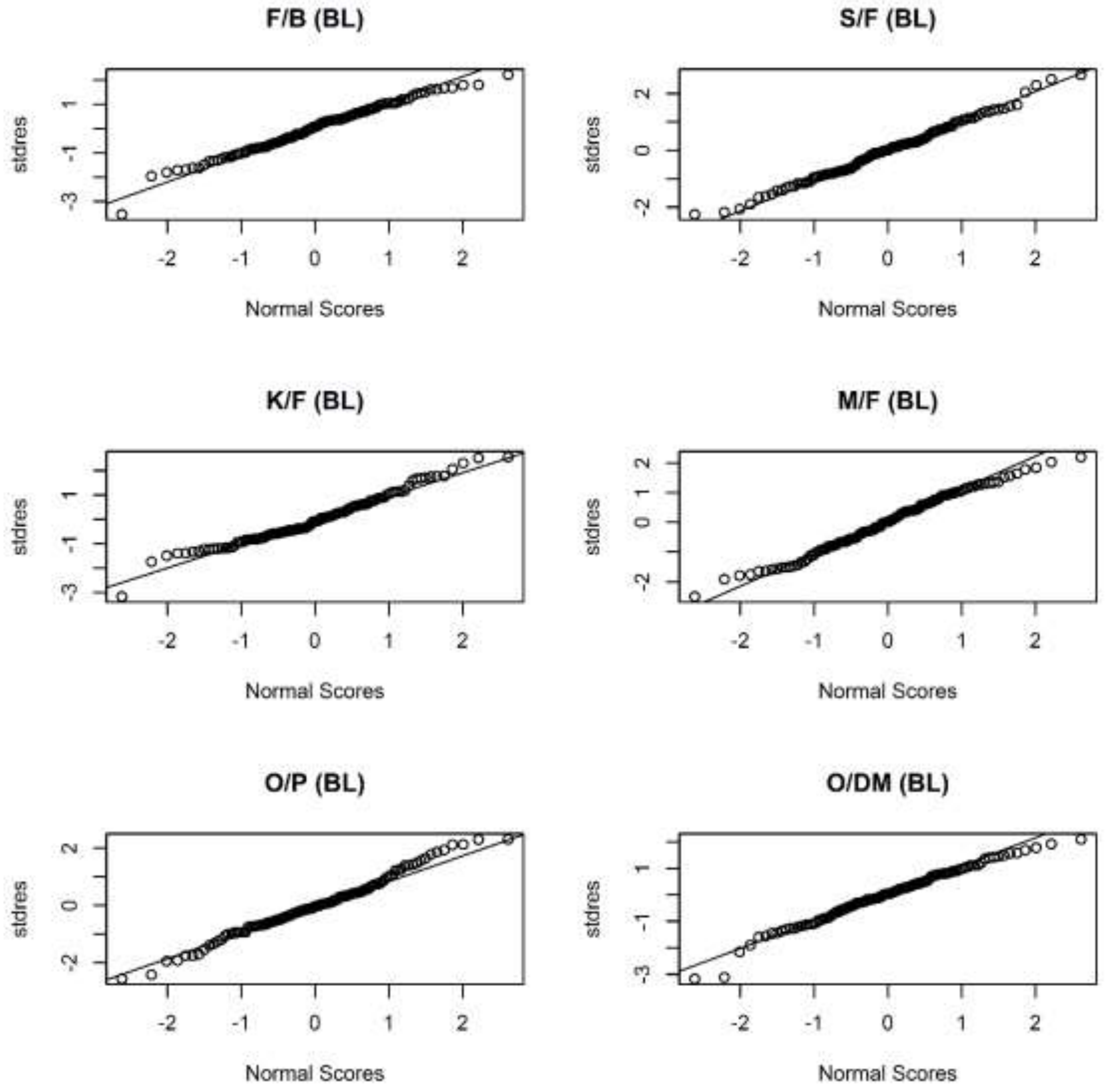

Supplementary Figure 4(f). QQ plots using BRR prediction models for all traits

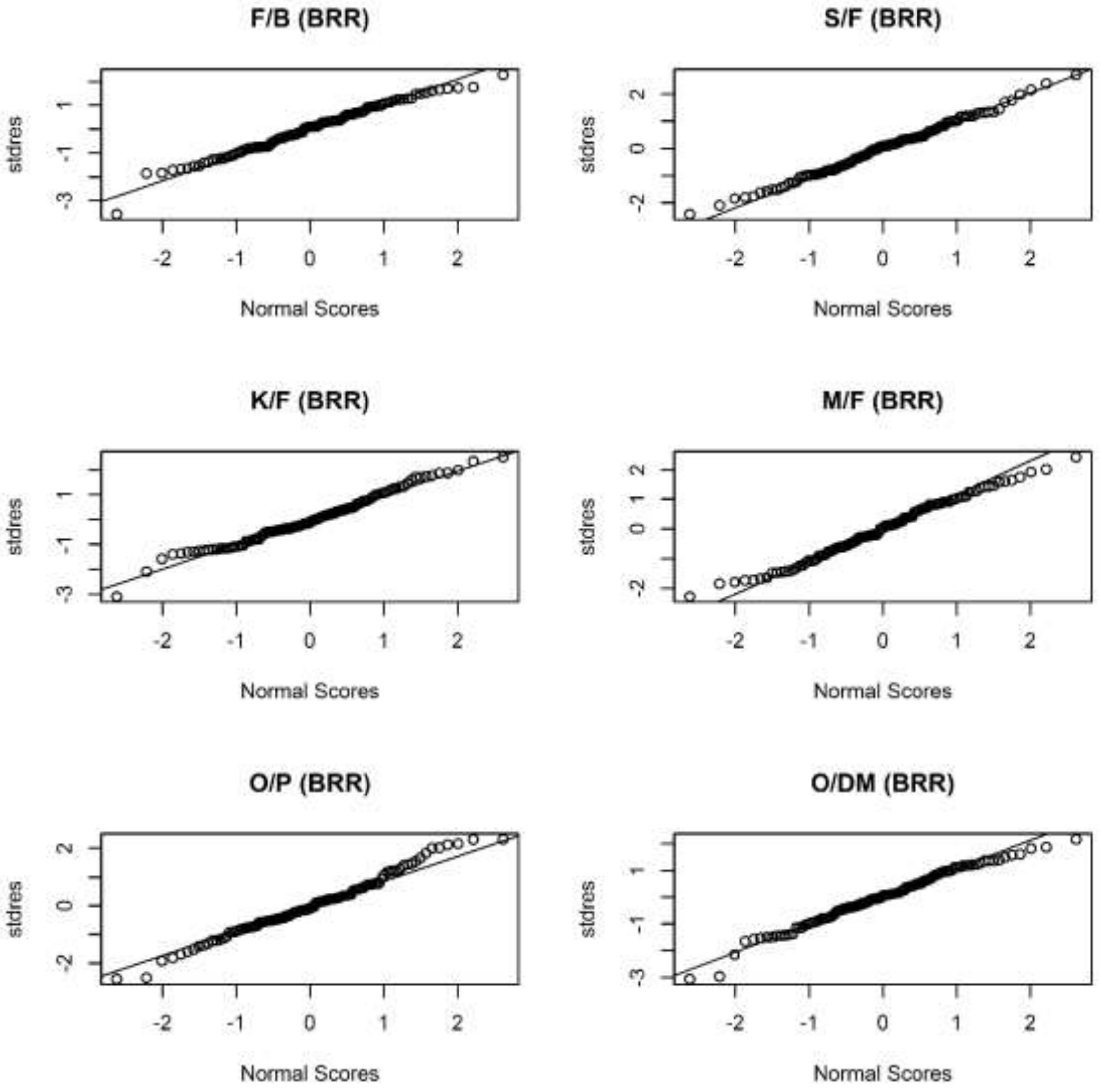

Supplement: Additional file 1: Figure S1. — Estimation of optimal MCMC iterations required for Bayes A - S/F and O/P. Figure S2. Distribution of kinship coefficients for 112 Deli x Nigerian individuals used in this study. Figure S3. Residual plots for different prediction models for all traits. Figure S4. QQ plots for different prediction models for all traits (PDF 640 kb) [file 12863_2017_576_MOESM1_ESM.pdf]
